# Determining health information needs among older adolescents (15–19 years): A survey in Geneva, 2023

**DOI:** 10.1016/j.pmedr.2026.103444

**Published:** 2026-03-10

**Authors:** Soroken Cindy, Baratti-Mayer Denise, Bonvin Nathalie, Morsa Maxime, Hariel Spinelli Anne-Laure

**Affiliations:** a Consultation des adolescents, Hôpitaux Universitaires de Genève, Boulevard de la Cluse 26, 1205 Genève, Switzerland; b Service de Santé de l’Enfance et de la Jeunesse, rue des Glacis-de-Rive 11, 1207 Genève, Switzerland; c Service de Santé de l’Enfance et de la Jeunesse, rue des Glacis-de-Rive 11, 1207 Genève, Switzerland; d Psychology Department, Adaptation, Resilience & Changement Unit of Research, University of Liege, Belgium; e Hôpitaux Universitaires de Genève, Maison de l’enfant et de l’adolescent, Boulevard de la Cluse 26, 1205 Genève, Switzerland

**Keywords:** Adolescents, Health information, Information needs, Youth consultation, Public health, Adolescent and young adult (AYA)

## Abstract

**Objective:**

To assess health information needs, preferred information sources, and willingness to engage in health promotion among older adolescents (15–19 years) in Geneva, Switzerland.

**Methods:**

Data were collected from 950 adolescents (aged 15–19 years) between October and November 2023 **in Geneva**, using a classroom-based online survey.

**Results:**

Adolescents expressed interest across a wide range of health topics, with Sleep receiving the highest average score. Girls expressed greater information needs in areas such as mental health, diet and self-perception, and interest generally increased with age. No significant differences were observed between public and private school students. Family remained the preferred source for diet, self-perception and safety/risk-taking, whereas social media was most used for environment, self-perception and social life. Overall, 32.6% of participants reported high readiness (scores 7–10) to engage in health promotion activities.

**Conclusions:**

One-third of adolescents indicated strong willingness to participate in health promotion, highlighting an opportunity to involve them directly in public health initiatives. Parents remained key and trusted sources of information, underscoring the need to support them with accurate knowledge and communication tools. Strengthening educational and communication strategies, particularly within primary care and school health services, can enhance adolescents' access to reliable information, support informed decision-making**,** and improve overall well-being.

## Highlights

• Health interests evolve with age during adolescence.
• Adolescents show a growing interest in a wide range of health topics.
• Families remain a key, trusted source of health information.
• Adolescents are ready to engage in health promotion.

## 1 Introduction

Adolescence is a critical developmental period characterized by rapid biological, cognitive, emotional, and social changes that shape lifelong health trajectories (WHO, 2025). During this stage, individuals develop autonomy, establish health-related behaviors, and seek information for decision-making.

In recent years, the ways adolescents access health information have evolved considerably, particularly with the expansion of digital media. While online platforms provide unprecedented access to information, they also expose young people to misinformation and variable-quality content (Suarez-Lledo and Alvarez-Galvez, 2021; Tonsaker et al., 2014). Previous studies suggest that adolescents rely on multiple sources, including family, peers, healthcare providers, and digital media, with notable gender and age-related differences in information-seeking behavior (Ettel et al., 2012; Martinović et al., 2023). However, relatively few studies have examined adolescents' self-identified health information needs across a broad range of topics.

A previous survey conducted in Geneva among adolescents aged 12–15 years identified key health information interests, including *Sleep*, *Self-perception*, *Biology*, *Mental health* and *Physical activity*, with notable gender differences in topic preferences *(*Hariel Spinelli et al., 2024*)*. That study also found that more than one-third of participants expressed willingness to engage in health promotion activities, highlighting adolescents' perceived importance of health information. Health information needs may evolve substantially across adolescence as cognitive maturity, social contexts, and autonomy increase (Saraipour et al., 2025; WHO, 2025). Understanding whether and how these needs differ in later adolescence is essential for tailoring prevention and health promotion strategies.

To better understand health information needs across adolescence, we conducted a separate study among older adolescents aged 15–19 years in Geneva, corresponding to the upper age range of the World Health Organization definition of adolescence (10–19 years) (WHO, 2025). Adolescents were consulted during the development and refinement of the survey instrument to ensure relevance and clarity.

The primary aim of this study was to assess health information needs, preferred information sources, and willingness to engage in health promotion among adolescents aged 15–19 years in Geneva. As a secondary objective, we explored similarities and differences with findings from our previous survey conducted among younger adolescents (12–15 years).

## 2 Methods

### 2.1 Study design and population

We conducted an exploratory cross-sectional quantitative survey in Geneva, Switzerland.

In this manuscript, the term “older adolescents” refers to participants aged 15–19 years.

*Adolescent consultation in survey development.*

A group of 7 adolescents (5 females and 2 males), aged 15–19 years from a public secondary school in Geneva participated in two consultation sessions held in May 2023. Participants were recruited on a voluntary basis. Their role was to review the questionnaire previously developed for the 12–15-year-old cohort and provide feedback regarding language clarity, comprehension, and age-appropriateness.

The consultation resulted in minor linguistic adjustments, including rewording of certain items to improve clarity and better reflect terminology used by older adolescents. No new thematic domains were introduced, and the overall structure and content of the questionnaire remained unchanged.

In August 2023, a pilot test was conducted with an additional group of 18 adolescents (5 females and 13 males) aged 15–19 years from a public secondary school to assess comprehensibility and completion time. Feedback from this phase led to minor wording refinements before finalizing the online version of the survey.

Adolescents were involved in the consultation and pilot phases of instrument refinement but were not engaged in data analysis, interpretation, or dissemination of findings.

*Population.*

Students from secondary II education in Geneva (aged 15 to 19) are offered two paths: the academic path, leading to the Swiss Maturity Certificate and university admission, and the vocational path, which combines theoretical education with practical training in a company. This system, equivalent to high school in the United States, provides diverse academic and vocational pathways.

To enhance diversity of socio-educational backgrounds, we recruited adolescents, regardless of their health status, from both public and private schools and from different educational pathways (academic and vocational). The Geneva secondary education system includes students from varied socio-economic contexts. No socio-economic exclusion criteria were applied.

Adolescents who were not able to read French were excluded, as the questionnaire was administered exclusively in French and no translated versions were available.

*Recruitment.*

The sample size calculation was based on the total number of students enrolled in upper secondary education (secondary II) in Geneva (28784), which corresponds predominantly to adolescents aged 15–19 years. In Switzerland, secondary II includes post-obligatory education for this age range and represents the target population from which the survey sample was drawn. The recommended minimum sample size (using a tool from the Survey Monkey® website) was of 589, with a confidence level of 95% and a margin of error of 4%.

In collaboration with the Department of Public Instruction of the Canton of Geneva, an information letter was sent at the end of summer 2023 to high schools' deans, inviting voluntary participation. Eight high schools were recruited (6 public and 2 private).

Participation was entirely voluntary. Students were informed that they could decline participation or discontinue the survey at any time without academic or personal consequences. The survey was completed anonymously, and no identifying information was collected. Students who chose not to participate were allowed to remain in the classroom and engage in alternative activities without penalty.

Teachers received written instructions and a standardized manual explaining how to administer the survey. They were present only to supervise classroom activities and did not monitor individual responses.

*Procedure.*

The survey was administered using Google Forms® due to its accessibility and familiarity among adolescents in the school setting. It was accessed anonymously via a QR code. No names, email addresses, login requirements or other identifying information were collected. The study did not collect clinical or medical data but focused on self-reported health information interests and preferences. Responses were accessible only to the research team and were downloaded for analysis.

It was distributed in French between October and November 2023. Students accessed the survey through the QR code, which led to a brief description of the study. Completion of the survey was considered implied consent.

The survey was administered during a dedicated one-hour class session (typically computer science courses identified as non-prejudicial to the curriculum). It was conducted in four Academic High Schools, one Business School, one Vocational Training Center for the Arts, one Specialized Vocational School, and one Vocational Training Center for Construction.

*Ethics.*

In Switzerland, the law on research with humans is focused on informed consent regarding disease and the normal functioning of the human body. Since this study does not concern disease, it was considered to fall outside the scope of Swiss human research legislation and received an exemption from the Swiss Ethics Committee. Nevertheless, we provided adolescents with a detailed explanation of the study, and they were given the time and freedom to decide whether to participate. They could at any time stop the survey.

### 2.2 Measures

We created a comprehensive 86-item questionnaire covering 15 topics: *Self-perception*, *Mental health*, *Addictions*, *Safety and Risk Taking*, *Sleep*, *Biology*, *Sexuality*, *Physical activity*, *Food*, *Pandemics*, *Violence*, *The Environment*, *Natural Medicine*, *Research* and *Social Aspects*. It was based on the instrument previously used among younger adolescents in Geneva. The overall structure and thematic domains were maintained to ensure comparability across age groups. A limited number of items were added or slightly reformulated to better reflect developmental differences in older adolescents, particularly regarding suicide and self-harm, help-seeking behaviors, denial of pregnancy, and communicable diseases. With the exception of these adaptations, the majority of items remained conceptually consistent, allowing meaningful comparison across most health domains.

In the questionnaire, each topic was introduced by a short explanatory statement. Participants were then asked to respond to the following prompt: “*In the field of […] I would like to be better informed about: […]*”. Responses were recorded on a four-point Likert scale ranging from 1 (*no*) to 4 (*yes*), with intermediate options “*rather no*” and “*rather yes*”.

A brief description of each topic and example items is provided in Table 1. The full questionnaire is available as Supplementary Material.

**Table 1:** Description of the 15 health information domains included in the questionnaire administered to adolescents aged 15–19 years in Geneva, Switzerland (2023).

**Topic**	**Brief Description**	**Number of Items**
Self-perception	Self-esteem, body image, assertiveness, self-confidence, self-acceptance	5
Mental health	Stress management, mood changes, mental disorders, prevention, suicide/self-harm, helping others	8
Addictions	Alcohol, tobacco, illicit drugs, screen use, access to help	5
Safety and risk-taking	Peer influence, accident response, risky driving, dangerous behaviors	4
Sleep	Sleep phases, hygiene recommendations, consequences of sleep deprivation, sleep disorders	4
Biology	Normal body functioning, illness mechanisms, recognizing warning signs	3
Sexuality	Puberty, attraction, gender identity, consent, contraception, STIs, pregnancy, abortion, pornography, legal aspects	14
Physical activity	Sedentary behavior, benefits of exercise, recommendations, excessive sport	4
Diet	Balanced nutrition, junk food risks, hunger cues, diets, intolerances, eating disorders, weight	7
Pandemics	Communicable diseases, hygiene, public health measures, vaccines, COVID-19	5
Violence	Physical, sexual, incest, bullying, cyber-harassment, consequences, response strategies	8
Environment	Pollution, climate change, eco-anxiety, prevention, future food systems	5
Alternative medicines	Alternative therapies, scientific evidence, complementarity with conventional medicine	3
Medical research	Research process, scientific achievements, misinformation, animal research	4
Social life	Socioeconomic factors, culture, disability, financial hardship, relationships	7

The full questionnaire is available as Supplementary Material.

The study was completed with the assessment of the sources of information used in general: Social media (Twitter/X, Facebook, Instagram, TikTok), Family, the Internet, Doctor, Friends, School nurse, Teachers / Educators and the source of information used for each topic.

Participants rated the importance of health promotion within their social circles on a scale of 1 to 10 (from “1 = *not at all important*” to “10 = *very important*”), as well as their readiness to engage in health promotion, using the same scale (from “1 = *not at all*” to “10 = *very ready*”).

Then the questioner ended with a few demographic questions (age, gender identity, the number of family members and educational paths).

### 2.3 Statistical analysis

Statistical analyses were conducted with the support of an external statistician. Statistical significance was set at *p* < 0.001.

We calculated summary scores for each of these topics based on the mean score of the relevant items (between 3 and 11 per topic) and obtained high internal consistency coefficients (Cronbach's alpha 0.80 to 0.96). We then used analysis of variance, equivalent to a *t*-test for two-group comparisons, to compare these scores between different subgroups, including girls vs. boys, different age groups (from 16 to 19, by year), and public vs. private school attendees. For age groups, we obtained a *P* value for the linear trend.

We obtained distributions of all 86 information content items and computed the average value (on a scale between 1 and 4) and the standard deviation.

Analyses were conducted using SPSS version 28 (IBM Corp., Armonk, NY, USA).

## 3 Results

We collected data from 950 respondents on their information interests; of whom 4 failed to answer any questions and were excluded from further analysis. Due to the survey design, we cannot determine how many adolescents were actually invited to participate.

Participants self-reported their ages as follows: 217 (23.5%) were under 16, 290 (31.5%) were 16 years old, 211 (22.9%) were 17, 100 (10.8%) were 18, 44 (4.8%) were 19, and 60 (6.5%) were over 19. A total of 24 participants did not report their age.

The majority attended public schools (725, 79.2%), while 190 (20.8%) went to private schools; thirty-one did not report school type.

Overall, 718 respondents (75.9%) completed all 86 questionnaire items. Additionally, 179 (18.9%) answered between 80 and 85 items, 43 (3.5%) completed between 40 and 79 items, and 16 (1.7%) responded to 1–39 items.

Regarding gender identity, 469 (51.1%) identified as female, 411 (44.8%) as male, 16 (1.7%) as nonbinary and 21 (2.3%) as other; 29 participants did not disclose their gender. Due to the small number of participants identifying as nonbinary or “other,” these subgroups were included in descriptive statistics but were not included in gender-based comparative analyses.

Across the 86 information items, mean scores ranged from 1.71 for *Covid-19* to 3.04 for *Stress management*, on a 1 (no) to 4 (yes) scale, with higher scores reflecting greater interest in receiving information.

For all 15 information topics, the corresponding items were moderately to strongly correlated, and as a result, the Cronbach's alpha coefficients were reasonably high (0.80 to 0.96; Table 2). This justified the computation of domain-specific summary scores. Item-level patterns were consistent with domain-level averages, suggesting that the observed findings were not driven by specific question wording. No dominant theme was identified. The highest average score was for *Sleep* (2.86, *p* < 0.001), followed by *Biology* and *Mental Health* and the lowest was for *Pandemics* (2.22, p < 0.001) (Table 2).

**Table 2:** Internal consistency (Cronbach's alpha) and mean scores of 15 domain-specific health information scales among adolescents aged 15–19 years (*n* = 946) in Geneva, Switzerland, 2023.

	N items	Missing, N	Cronbach alpha	Mean⁎ (standard deviation)
Perception of self	5	28	0.92	2.59 (0.89)
Mental health/well-being	8	56	0.91	2.70 (0.81)
Addictions	5	33	0.87	2.33 (0.93)
Safety and risk-taking	4	38	0.80	2.44 (0.80)
Sleep	4	34	0.86	2.86 (0.87)
Biology	3	37	0.85	2.73 (0.89)
Sexuality/reproduction	14	67	0.96	2.54 (0.91)
Physical activity	4	37	0.90	2.66 (0.94)
Diet	7	50	0.89	2.65 (0.85)
Pandemics	5	45	0.86	2.22 (0.87)
Violence	8	54	0.94	2.58 (0.96)
Environment	5	36	0.91	2.58 (0.96)
Alternative medicines	3	42	0.92	2.53 (1.03)
Medical research	4	49	0.87	2.44 (0.93)
Social life	7	51	0.93	2.59 (0.92)

⁎ : Scale ranging from 1 (no) to 4 (yes).

Girls expressed generally somewhat higher needs for information than boys (Table 3) with the most marked differences (p < 0.001) concerning *Self-perception*, *Mental Health*, *Sexuality/Reproduction*, *Diet*, *Violence*, *Environment*, *Alternative Medicines* and *Social Life*.

**Table 3:** Comparison of mean domain-specific health information scores by gender, age group, and school type among adolescents aged 15–19 years (n = 946) in Geneva, Switzerland, 2023.

	**Gender**			**Age (years)**					**School system**		
	**Boys**	**Girls**	**p**	**<16**	**16**	**17**	**≥18**	**p**⁎	**Public**	**Private**	**p**
**Number**	409	469		217	289	211	204		724	190	
**Perception of self**	2.42	2.74	<0.001	2.47	2.65	2.54	2.71	0.03	2.60	2.56	0.59
**Mental health/well-being**	2.50	2.90	<0.001	2.50	2.75	2.74	2.83	<0.001	2.69	2.76	0.33
**Addictions**	2.28	2.37	0.14	2.23	2.35	2.33	2.43	0.05	2.32	2.39	0.36
**Safety and risk-taking**	2.36	2.53	0.00	2.32	2.47	2.42	2.58	0.00	2.45	2.44	0.97
**Sleep**	2.78	2.96	0.00	2.64	2.86	2.95	3.05	<0.001	2.88	2.82	0.39
**Biology**	2.66	2.83	0.01	2.54	2.77	2.79	2.85	<0.001	2.76	2.66	0.16
**Sexuality/reproduction**	2.33	2.73	<0.001	2.29	2.59	2.62	2.69	<0.001	2.53	2.60	0.33
**Physical activity**	2.72	2.62	0.12	2.54	2.59	2.70	2.84	<0.001	2.66	2.65	0.85
**Diet**	2.52	2.77	<0.001	2.49	2.62	2.66	2.86	<0.001	2.67	2.57	0.13
**Pandemics**	2.20	2.24	0.48	2.01	2.27	2.24	2.37	<0.001	2.23	2.18	0.49
**Violence**	2.30	2.85	<0.001	2.27	2.64	2.77	2.65	<0.001	2.59	2.59	0.97
**Environment**	2.45	2.73	<0.001	2.34	2.60	2.61	2.78	<0.001	2.60	2.53	0.40
**Alternative medicines**	2.40	2.65	<0.001	2.27	2.52	2.64	2.68	<0.001	2.52	2.55	0.77
**Medical research**	2.37	2.54	0.01	2.22	2.48	2.51	2.58	<0.001	2.47	2.38	0.27
**Social life**	2.41	2.77	<0.001	2.33	2.60	2.70	2.75	<0.001	2.60	2.57	0.66

⁎ : P value for linear trend across age groups.

Information needs increased with age for all items, but not statistically significantly for three (*Addictions*, *Safety and Risk-taking* and *Self-perception*). No statistically significant differences were observed between public and private schools regarding information needs (Table 3).

Regarding the preferred sources of information used, 144 respondents (15.2%) did not select any source, whereas 352 respondents (37.2%) selected all seven suggested sources. The distribution of preferred information sources across the different health domains is presented in Table 4.

**Table 4:** Preferred sources of health information by domain (percentages) among adolescents aged 15–19 years (n = 946) in Geneva, Switzerland, 2023.

	**Social media**	**Family**	**Internet**	**Doctor**	**Friends**	**Teachers**	**School nurse**	**None**
Perception of self	21.8	26.3	3.5	1.4	13.0	0.6	0.7	32.7
Mental health/well-being	20.1	19.9	13.1	5.5	10.5	1.1	1.1	28.9
Addictions	19.7	15.4	13.8	3.5	7.9	2.7	1.3	35.6
Safety and risk-taking	11.8	23.7	9.9	2.2	5.5	4.4	1.2	41.2
Sleep	15.5	23.3	15.8	7.5	3.9	1.4	1.3	31.4
Biology	10.5	10.7	15.3	3.6	1.9	16.8	0.8	40.4
Sexuality/reproduction	19.5	12.2	11.4	2.7	11.1	3.0	2.1	38.1
Physical activity	18.5	20.9	10.9	4.1	5.8	3.9	0.6	35.2
Diet	17.1	27.9	11.8	4.5	3.4	1.3	1.0	33.0
Pandemics	16.8	14.2	16.6	3.4	2.0	2.7	1.5	42.8
Violence	21.2	15.8	10.6	1.3	6.3	1.9	1.1	41.9
Environment	22.4	13.6	12.7	1.2	4.0	5.9	1.0	39.2
Alternative medicines	13.8	16.1	12.6	4.5	2.9	2.2	1.3	46.6
Medical research	13.4	12.1	15.4	5.1	1.8	5.1	1.2	46.0
Social life	21.2	19.1	7.1	1.2	9.4	1.2	1.1	39.7

Family members were the preferred source of information for topics such as *Diet*, *Self-perception*, *Safety and Risk-taking*, *Sleep*, *Physical Activity* and *Alternative Medicines*. Social media was most frequently used for information on *Environment*, *Violence*, *Social Life*, *Mental Health*, *Addictions*, *Sexuality/Reproduction* and *Pandemics*. Family and social media showed similar usage results for *Mental Health/Well-being*, *Biology* and *Medical Research*. Internet was the preferred source for *Medical Research*, while teachers were favored for biology-related information. Over 40% of participants did not report using any of the proposed sources for *Safety and Risk-taking*, *Violence*, *Biology*, *Pandemics*, *Alternative Medicine* and *Medical Research*. No information source exceeded 27.9%.

In the subgroup comparisons, significant gender differences were observed only for *Self-perception* and *Mental Health*. In these topics, girls were more likely than boys to prioritize social media as their preferred source of information. No significant differences in preferred sources of information were found across age groups.

With regard to the question “*How important do you think it is to promote health in your social circle?*”, the average score was 6.8, with a standard deviation of 2.7. 59.7% of respondents rated health promotion as important to very important (7–10 on the scale). Girls had higher scores than boys, and older students had higher scores than younger students. No difference by type of school.

As for the question: “*To what extent do you feel ready to participate in health promotion actions?*”, the average score was 5.0, with a standard deviation of 2.8. The proportion of participants scoring between 7 and 10 was 32.6%. Girls had higher scores than boys, and private school students had higher scores than public school students. No difference was identified by age.

## 4 Discussion

The survey achieved a high completion rate, with most adolescents responding to the majority of items. Across the age range, no single health topic dominated; rather, adolescents expressed interest in a broad spectrum of themes, with a tendency toward increasing interest with age. This pattern may reflect developmental changes in autonomy and cognitive maturation during late adolescence, as suggested in previous developmental research.

*Sleep* and *Mental Health* generated the highest levels of interest. Sleep plays a central role in adolescent cognitive, emotional, and behavioral functioning (Tarokh et al., 2016) and sleep disturbances are increasingly reported in many countries (Ghekiere et al., 2019). Mental health similarly emerged as a priority, consistent with global data indicating that many mental disorders begin during adolescence (Solmi et al., 2022). These findings support the importance of strengthening accessible and age-appropriate mental health and sleep-related health education initiatives. Interest in *Biology* was also strong, possibly reflecting developmental curiosity about bodily changes and health.

Regarding information sources, our findings highlight a dual pattern. Family remained a key and trusted source for several lifestyle-related topics (Känsäkoski et al., 2021; Martinović et al., 2023), while social media and online platforms were more frequently used for psychosocial and societal issues. Parental influence appears to persist into late adolescence, as family communication patterns and emotional support shape how young people interpret and apply health information (Steinberg and Duncan, 2002). Supporting parents with accurate knowledge and communication tools may therefore enhance the effectiveness of adolescent health promotion strategies (Cohen et al., 2025). Healthcare providers (including school health professionals) were less frequently selected. These patterns align with prior research showing adolescents' preference for accessible and confidential information channels, particularly for sensitive topics (Cunningham et al., 2009; Katavić et al., 2020).

Although digital platforms offer convenience, the growing availability of online information raises concerns about misinformation and uneven content quality (Suarez-Lledo and Alvarez-Galvez, 2021; Tonsaker et al., 2014). Strengthening digital health literacy and promoting reliable, adolescent-oriented online resources remain important public health priorities (Taba et al., 2022). At the same time, improving communication between adolescents and healthcare providers and clarifying issues of confidentiality may help reduce barriers to professional consultation (Chung et al., 2024; Gazibara et al., 2020; WHO, 2025).

A notable proportion of adolescents reported using multiple information sources, which may suggest active comparison behaviors (Ettel et al., 2012). Conversely, a smaller subgroup did not report using any of the proposed sources, highlighting the challenge of reaching adolescents who may be less engaged in health information seeking (Kim, 2015; Martinović et al., 2023).

Gender differences were observed, consistent with existing literature (Ackard and Neumark-Sztainer, 2001; Martinović et al., 2023), with girls showing more interest in *Sleep*, *Mental Health*, *Violence* and *Social Life*, as well as differing source preferences for topics like *Self-perception* and *Mental Health*.

Comparison with the previous survey conducted among younger adolescents revealed substantial continuity in priority topics, particularly *Sleep* and *Mental Health*, while some differences reflected developmental shifts in interests: younger adolescents expressed comparatively greater interest in *Self-perception* and *Sexuality*. These findings underscore the importance of tailoring health promotion strategies to adolescents' evolving developmental stages (WHO, 2025).

As the survey was conducted in 2023, it should be noted that the digital information landscape continues to evolve rapidly, particularly with the expansion of generative artificial intelligence tools and algorithm-driven content platforms (McBain et al., 2025). Adolescents' health information needs and source preferences may therefore shift over time. Ongoing monitoring will be necessary to understand how emerging technologies influence adolescents' health information access.

Several limitations should be considered when interpreting these findings. Our presence during the working sessions may have influenced adolescents' spontaneity and, consequently, their responses. Additionally, our adolescent partners and participating schools were volunteers, which could introduce selection bias and limit the generalizability of our findings to the wider adolescent population. The exclusion of non-French-speaking adolescents may limit generalizability, particularly in a multilingual context such as Geneva.

## 5 Conclusion

Understanding adolescents' health information needs is essential for designing effective and developmentally appropriate health promotion strategies. Our findings show that older adolescents express broad and evolving interests across multiple health domains, with family remaining a central and trusted source of information.

Strengthening communication strategies, both within families and across primary care and school health settings, may enhance adolescents' access to reliable health information. Importantly, the willingness expressed by many participants to engage in health promotion initiatives represents a valuable opportunity to involve adolescents more actively in public health efforts.

## CRediT authorship contribution statement

**Soroken Cindy:** Writing – review & editing, Writing – original draft, Methodology, Investigation, Formal analysis, Conceptualization. **Baratti-Mayer Denise:** Writing – review & editing, Methodology, Conceptualization. **Bonvin Nathalie:** Writing – review & editing, Methodology, Conceptualization. **Morsa Maxime:** Writing – review & editing, Supervision, Methodology, Conceptualization. **Hariel Spinelli Anne-Laure:** Writing – review & editing, Validation, Supervision, Methodology, Investigation, Formal analysis, Conceptualization.

## Funding

This research did not receive any specific grant from funding agencies in the public, commercial, or not-for-profit sectors.

## Declaration of competing interest

The authors declare that they have no known competing financial interests or personal relationships that could have appeared to influence the work reported in this paper.

## Data Availability

Data will be made available on request.

## Appendix A Supplementary data

Supplementary material: Full questionnaire administered to adolescents aged 15-19 years in Geneva, Switzerland (2023) - English Version Image 1

## References

[bb0005] Ackard D.M., Neumark-Sztainer D. (2001). Health care information sources for adolescents: age and gender differences on use, concerns, and needs. J. Adolesc. Health.

[bb0010] Chung R.J., Lee J.B., Hackell J.M., Alderman E.M., COMMITTEE ON ADOLESCENCE, COMMITTEE ON PRACTICE & AMBULATORY MEDICINE (2024). Confidentiality in the care of adolescents: policy statement. Pediatrics.

[bb0015] Cohen K.S., Fallik D., Cappola A.R. (2025). The role of family as a source of health information among college students. J. Community Health.

[bb0020] Cunningham S.D., Kerrigan D.L., Jennings J.M., Ellen J.M. (2009). Relationships between perceived STD-related stigma, STD-related shame and STD screening among a household sample of adolescents. Perspect. Sex. Reprod. Health.

[bb0025] Ettel G., Nathanson I., Ettel D., Wilson C., Meola P. (2012). How do adolescents access health information? And do they ask their physicians?. Perm. J..

[bb0030] Gazibara T., Cakic J., Cakic M., Grgurevic A., Pekmezovic T. (2020). Searching for online health information instead of seeing a physician: a cross-sectional study among high school students in Belgrade, Serbia. Int. J. Public Health.

[bb0035] Ghekiere A., Van Cauwenberg J., Vandendriessche A., Inchley J., Gaspar de Matos M., Borraccino A., Gobina I., Tynjälä J., Deforche B., De Clercq B. (2019). Trends in sleeping difficulties among European adolescents: are these associated with physical inactivity and excessive screen time?. Int. J. Public Health.

[bb0040] Hariel Spinelli A.-L., Maxime M., Denise B.-M., Cindy S., Nathalie B. (2024). Determining adolescent health information needs: a survey in Geneva, 2022. Prev. Med. Rep..

[bb0045] Känsäkoski H.M., Hirvonen N., Palmgren-Neuvonen L., Nygård T., Huotari M.-L. (2021). Finnish adolescents' selection and assessment of health information sources. IR.

[bb0050] Katavić S.S., Martinović I., Kim S.U. (2020). College students' sexual health information needs and source preferences in relation to worry about sexual health outcomes. IR.

[bb0055] Kim S. (2015). An exploratory study of inactive health information seekers. Int. J. Med. Inform..

[bb0060] Martinović I., Kim S.U., Stanarević Katavić S. (2023). Study of health information needs among adolescents in Croatia shows distinct gender differences in information seeking behaviour. Health Info. Libr. J..

[bb0065] McBain R.K., Bozick R., Diliberti M., Zhang L.A., Zhang F., Burnett A., Kofner A., Rader B., Breslau J., Stein B.D., Mehrotra A., Pines L.U., Cantor J., Yu H. (2025). Use of generative AI for mental health advice among US adolescents and young adults. JAMA Netw. Open.

[bb0070] Saraipour, F., Panahi, S., Nemati-Anaraki, L., Shahraki-Mohammadi, A., 2025. Adolescents' health information-seeking behavior: a scoping review. J. Adolesc. Health S1054-139X(25)00240-X. doi:10.1016/j.jadohealth.2025.05.030. 40767803

[bb0075] Solmi M., Radua J., Olivola M., Croce E., Soardo L., Salazar de Pablo G., Il Shin J., Kirkbride J.B., Jones P., Kim J.H., Kim J.Y., Carvalho A.F., Seeman M.V., Correll C.U., Fusar-Poli P. (2022). Age at onset of mental disorders worldwide: large-scale meta-analysis of 192 epidemiological studies. Mol. Psychiatry.

[bb0080] Steinberg L., Duncan P. (2002). Work group IV: increasing the capacity of parents, families, and adults living with adolescents to improve adolescent health outcomes. J. Adolesc. Health.

[bb0085] Suarez-Lledo V., Alvarez-Galvez J. (2021). Prevalence of health misinformation on social media: systematic review. J. Med. Internet Res..

[bb0090] Taba M., Allen T.B., Caldwell P.H.Y., Skinner S.R., Kang M., McCaffery K., Scott K.M. (2022). Adolescents' self-efficacy and digital health literacy: a cross-sectional mixed methods study. BMC Public Health.

[bb0095] Tarokh L., Saletin J.M., Carskadon M.A. (2016). Sleep in adolescence: physiology, cognition and mental health. Neurosci. Biobehav. Rev..

[bb0100] Tonsaker T., Bartlett G., Trpkov C. (2014). Health information on the internet: gold mine or minefield?. Can. Fam. Physician.

[bb0105] WHO (2025). Adolescent health [WWW Document]. https://www.who.int/health-topics/adolescent-health.

