# Supplementary material for: Determining health information needs among older adolescents (15–19 years): A survey in Geneva, 2023
Source: Prev Med Rep. 2026 Mar 10;64:103444. doi: 10.1016/j.pmedr.2026.103444 (PMC12996190; doi:10.1016/j.pmedr.2026.103444)
Supplement: Supplementary file 1 — Supplementary material: Full questionnaire administered to adolescents aged 15-19 years in Geneva, Switzerland (2023) - English Version [file mmc1.docx]

Determining health information needs among older adolescents (15-19 years): A survey in Geneva, 2023

# Supplementary File 1 – Full Questionnaire (English Version)

This supplementary file contains the complete questionnaire administered to adolescents aged 15–19 years in Geneva (October–November 2023).

## Introduction

With this questionnaire, we aim to assess adolescents’ needs for information across different health domains.

For each topic below, please indicate how much you would like to receive information.

Response scale for Questions 1–15:

No / Rather no / Rather yes / Yes

## 1. Self-Perception

Self-esteem is a person's perception of their own worth and their ability to value their qualities and progress, according to the World Health Organization (WHO). Good self-esteem helps develop a sense of security and promotes a positive self-image. Regarding the perception I have of myself, I would like to be more informed about:

- Self-confidence
- Assertiveness
- Self-acceptance (differences, disability, etc.)
- Body image
- Self-esteem

## 2. Mental Health

Mental health is a state of well-being in which a person is able to overcome life's problems, be productive and relate well to others. In the field of mental health, I would like to be more informed about:

- Importance of social relationships for health
- Managing stress and pressure
- Normal mood changes and when to seek help
- Mental illnesses (depression, PTSD, bipolar disorder, schizophrenia, etc.)
- Treatment of mental illness
- Prevention and early warning signs
- Suicidal ideation and self-harm
- How to help someone who is suffering

## 3. Addictions

According to the WHO, addiction is a state of short- or long-term dependence on substances (alcohol, tobacco, etc.) or behaviours (games, screens, etc.). In the field of addictions, I would like to be more informed about:

- Risks of excessive alcohol consumption
- Risks of tobacco use (cigarettes, e-cigarettes, vaping)
- Risks of illicit substances (cannabis, cocaine, LSD, etc.)
- Risks of excessive screen use
- Where to find help in case of addiction

## 4. Safety and Risk-Taking

This theme concerns behaviors when individuals are perpetrators, witnesses or victims of dangerous situations. In the field of safety and risk-taking, I would like to be more informed about:

- Influence of family and peers on risky behavior
- What to do in case of an accident (first aid)
- Risks of dangerous driving
- Risks of dangerous physical stunts or street behavior

## 5. Sleep

This theme concerns sleep in general, its disorders and disruptors. In the field of sleep, I would like to be more informed about:

- Sleep phases
- Recommendations for healthy sleep
- Consequences of sleep deprivation
- Sleep disorders and their causes

## 6. Biology

This theme concerns the functioning of the human body when healthy or sick. In the field of biology, I would like to be more informed about:

- Body functioning when healthy
- Body functioning when ill
- Recognizing abnormal bodily signs

## 7. Sexuality

Sexuality is a central aspect of human life including intimacy, desire and identity. In the field of sexuality, I would like to be more informed about:

- Puberty
- Romantic and/or sexual attraction
- Gender identity
- Sexual relationships
- Consent and respect
- Sexual violence
- Contraception methods
- Emergency contraception
- Pregnancy
- Denial of pregnancy
- Abortion
- Sexually transmitted infections
- Pornography
- Legal aspects related to sexuality

## 8. Physical Activity

This theme concerns the health effects of physical movement. In the field of physical activity, I would like to be more informed about:

- Risks of sedentary behavior
- Health benefits of exercise
- Daily activity recommendations
- Risks of excessive sport

## 9. Diet

This topic includes everything related to food and nutrition. In the field of diet, I would like to be better informed about:

- Balanced nutrition
- Risks of ultra-processed foods
- Recognizing hunger and satiety signals
- Diet types
- Food intolerances and allergies
- Eating disorders
- Healthy body weight

## 10. Pandemics

A pandemic is the global spread of a new disease. In the field of pandemics, I would like to be more informed about:

- Communicable diseases
- Hygiene measures
- Public health measures
- Vaccines
- COVID-19

## 11. Violence

This theme refers to all forms of violence. In the field of violence, I would like to be better informed about:

- Physical or verbal violence
- Sexual violence
- Incest
- Bullying
- Cyber-harassment
- Consequences of violence
- How to react as victim, witness, or perpetrator

## 12. Environment

This theme concerns the impact of environmental factors on health. In the field of the environment, I would like to be better informed about:

- Pollution and pesticides
- Climate change
- Eco-anxiety
- Preventing environmental degradation
- Future food systems

## 13. Natural Medicine

This theme includes non-conventional therapeutic approaches. In the field of natural medicine, I would like to be more informed about:

- Alternative therapies
- Scientific evidence
- Complementarity with conventional medicine

## 14. Medical Research

This theme focuses on research aimed at improving health. In the field of research, I would like to be better informed about:

- How research is conducted
- Research achievements
- Reliable vs false information
- Research on animals

## 15. Social Determinants of Health

This theme relates to social and economic influences on health. In the social field, I would like to be better informed about:

- Socioeconomic influences
- Financial hardship and health
- Culture and health
- Illness/disability and social life
- Caring for someone ill/disabled
- Interacting with people with disabilities
- Building healthy relationships

## 16. Sources of Information (Multiple Choice)

Which sources do you use? (Multiple answers possible)

- TikTok
- Instagram
- Facebook
- Twitter
- Podcasts / YouTubers
- Institutional websites
- Non-institutional websites
- Printed media
- Parents
- Friends
- School nurse
- Siblings
- Family physician
- Teachers / educators

## 17. Preferred Source by Topic

Options: Social media; Family; Websites; Family physician; Friends; Teachers/educators; School nurse.

## 18. Readiness to Participate in Health Promotion

Scale 1–10 (1 = Not at all ready; 10 = Fully ready).

## 19. Perceived Importance of Health Promotion

Scale 1–10 (1 = Not important; 10 = Very important).

## 20. Top 3 Preferred Topics (Ranking)

Participants selected their three favorite themes in order of preference.

## 21. Open Question

What other health topic(s) would you like to receive information about?

## 22–26. Sociodemographic Information

- Age
- Type of training
- Type of institution (public/private)
- Gender identity
- Number of people living at home
